# Micro‐ or macroscale? Which one best predicts the establishment of an endemic Atlantic Forest palm?

**DOI:** 10.1002/ece3.5300

**Published:** 2019-06-02

**Authors:** Gabriela Akemi Macedo Oda, Marinez Ferreira de Siqueira, Alexandra dos Santos Pires, Rita de Cássia Quitete Portela

**Affiliations:** ^1^ Botanical Garden Research Institute of Rio de Janeiro – JBRJ Rio de Janeiro Brazil; ^2^ Department of Environmental Sciences, Forestry Institute Federal Rural University of Rio de Janeiro – UFRRJ Seropédica Brazil; ^3^ Department of Ecology, Institute of Biology Federal University of Rio de Janeiro ‐ UFRJ Rio de Janeiro Brazil; ^4^ Present address: Department of Environmental Sciences, Forestry Institute Federal Rural University of Rio de Janeiro – UFR BR465, Km7, 23897‐970 Seropédica RJ Brazil

**Keywords:** ecological niche modeling, germination, predation, regeneration ecology, sowing experiment, *Syagrus weddelliana*

## Abstract

Historically, macroecology and microecology have diverged with regard to the niche concept. A better understanding of functioning ecological systems, however, depends on an integrative approach to this concept at different spatial scales. A mixed approach, merging macro‐ and microscale by validating ecological niche modeling (ENM) with the results of in situ experiments and environmental data collection was used to understand if areas identified by ENM as highly suitable for adult palms are also adequate for seedling establishment. *Syagrus weddelliana*'s (Arecaceae) distribution range falls within the Atlantic Rain Forest, and more specifically Serra dos Órgãos region (Rio de Janeiro state), southeastern Brazil. The following steps were performed: (a) ENM to delimit the area of occurrence of *S. weddelliana* and locate experimental areas; (b) a seed sowing experiment in areas with presence or absence of the species in areas of high or low environmental suitability at 36 experimental stations; and (c) characterization of each microhabitat which was related back to the macroscale results of ENM. Evidence of biotic and abiotic limitations was found for *S. weddelliana* distribution. Areas of higher suitability had lower seed predation rates and, consequently, higher seed germination rates. On the other hand, areas with low environmental suitability at the macroscale were divided into two types: areas with microhabitat similar to that of areas with high environmental suitability that had some germination despite high predation and areas with different environmental conditions that had no germination and high predation rates. Seedlings and adults had different abiotic requirements. Microhabitat conditions were more important for the initial establishment of *S. weddelliana* than macroclimatic variables. This finding demonstrates that macro‐ and microecological information works in a complementary way to a better understanding of the distribution of *S. weddelliana*.

## 1 INTRODUCTION

Many concepts and theories in ecology are highly debated, often with divergent ideas among the different branches of ecology. Macroecology and experimental ecology, for example, have historically diverged with regard to the concept of the niche. While the first conceptualizes the niche as a space unit, based on environmental conditions (Grinnell's concept, 1917), the second defines it as the sum of all physical and biological variables acting in a community (Hutchinson's concept, 1957). Recent studies, however, have demonstrated the necessity of integrating both concepts and both spatial scales to better understand the functioning of ecological systems (Peterson et al., 2011; Soberon, 2007; Staniczenko, Sivasubramaniam, Suttle, & Pearson, 2017).

Ecological niche modeling (ENM) is one of the most widely used methods in macroecology for the evaluation of environmental similarity between regions and, consequently, to estimate species distributions (Peterson & Soberon, 2012). It is also an important tool used in the development of management and conservation strategies, with applications ranging from identification and detailing of biophysical characteristics of a given species’ habitat to the detection of environmental changes (Anderson, Lew, & Peterson, 2003; Peterson et al., 2011).

Despite its utility, ENM has its shortcomings. Besides uncertainty about input data and algorithms (Elith & Leathwick, 2009; Wiens, Stralberg, Jongsomjit, Howell, & Snyder, 2009), ENM is also limited in its use at small spatial scales since the minimum scale used for most input data is 1 Km. Some plant species, for example, have such narrow regenerative niches that slight environmental variation due to topography (Svenning, 2001) and edaphic and hydrological characteristics can limit their occurrence (John et al., 2007; Tuomisto et al., 1995). In these cases, in situ data obtained at small scales can provide a more refined view of the environment and help delimit the distribution of species. This is especially important for species with restricted distributions and specific microhabitat requirements, which are more vulnerable to habitat loss and, consequently, population declines or even extinction (O'Grady et al., 2004).

Another limitation of ENM is that it considers only environmental factors as determinants of species’ distribution (Eiserhardt, Svenning, Kissling, & Balslev, 2011; Lambers, Chapin, & Pons, 1998). For plants, important biotic factors can also affect species establishment, such as those involved in seed dispersal and predation processes that begin to operate upon the arrival of a propagule to a new place until its germination (Eiserhardt et al., 2011). Seed germination marks the beginning of the life cycle of an individual plant and is the first major life‐history transition (2015, & Nicotra, 2^2015^). Thus, there is strong selection pressure on germination strategies and timing to guarantee seedling emergence in appropriate environmental conditions for survival and growth (Donohue, Casas, Burghardt, Kovach, & Willis, 2010; Spindelböck et al., 2013).

This study employed a mixed approach, merging macro‐ and microscale, to understand if areas of high suitability for adult plants, identified through ENM, are also adequate for initial plant establishment. An Atlantic Forest palm with a narrow distribution within this biome was used as a study model. An ecological niche model was generated to delimit the area of occurrence for *Syagrus weddelliana* (H.Wendl.) Becc. and to locate experimental areas, and a sowing experiment was carried out along an environmental suitability gradient to assess initial establishment. Finally, microhabitat was characterized and related back to the macroecological results of ENM.

## 2 METHODS

### 2.1 Species and study area

*Syagrus weddelliana (H.Wendl.) Becc*. (1916) is a single‐stemmed palm with stems that are 1–5 m in height and up to 10 cm in diameter. Its fruits are brown, ovoid to ellipsoid, 1.7–2.3 cm long, and 1.0–1.7 cm in diameter, and with a homogeneous endosperm (Henderson, 2009). The species is endemic to the Atlantic Forest and has a narrow geographic distribution in the Serra dos Órgãos region (Rio de Janeiro state), southeastern Brazil (Leitman, Henderson, Noblick, & Martins, 2012), where it occurs on steep slopes usually below 1,000 m a.s.l. (Henderson, 2009). This area harbors three important conservation units where data were collected: Ecological Station of Paraíso (ESP, 4,930 ha, 22°27′ S 42°50′ W), Três Picos State Park (TPSP, 65,113 ha, 22º27′ S 42º46′ W), and Serra dos Órgãos National Park (SONP, 20,030 ha, 22º54′ S 42º09′ W).

The region has a hilly terrain, wide variation in elevation, and extends from sea level to 2,263 m. It contains various plant formations of the Atlantic Forest biome that vary from submontane ombrophilous dense forest to “campos de altitude” (high altitude grasslands) (ICMBio, 2008). At lower elevations, which correspond to the base of the mountain range, the climate is hot and humid with no dry season, corresponding to Koeppen's Af classification (Koeppen, 1948). The slopes and the top of the mountain range exhibit Cfa and Cfb climate types, with humid and mesothermic conditions and gradually decreasing temperature with increasing elevation (Bernardes, 1952). Annual average rainfall ranges from 1,500 to 3,000 mm, and average temperatures range from 18 to 26°C, with temperatures below 0°C occasionally observed at higher elevations (ICMBio, 2008).

### 2.2 Ecological Niche Modeling (ENM)

In 2012, data were collected during exploratory field trips to target areas with an attempt to cover the entire region of occurrence. After 10 expeditions, 14 points of occurrence were obtained, separated from each other by a minimum distance of 1 km. In addition, two of the four points provided in Species Link, GBIF database (http://www.splink.org.br/; https://www.gbif.org/), were used after checking geographic coordinates and eliminating inconsistent information. Thus, a total of 16 points of occurrence for *S. weddelliana* were used.

The 19 bioclimatic variables of WorldClim (http://www.worldclim.org/current) and the 17 topographic variables of the U.S. Geological Survey (http://edcdaac.usgs.gov/gtopo30/hydro/) were analyzed. Four variables were selected using principal component analysis (PCA) results and based on knowledge about their importance to species physiology and ecology: isothermality (mean daily temperature variation/annual thermal amplitude) (Bio 3), temperature seasonality (Bio 4), annual precipitation (Bio 12), and slope. All the variables used were numerical and continuous with 30 s (≈1 km) resolution.

The ecological niche model was estimated using the combination of four algorithms: Bioclin, SVM, Randon forest, and Maxent. The model was set up to perform three replications of the original data set using two partitions of the occurrence points for training the model and one for testing. The training and test sets were chosen randomly from the total set of points, using a data reset at each selection, following the KFold partition method (Fielding & Bell, 1997). For model evaluation, we calculated the true skill statistic (TSS) (Allouche, Tsoar, & Kadmon, 2006), which quantifies the ability of a model to correctly classify presences and absences (calculated as sensitivity + specificity − 1) for a threshold that, in this case, maximizes sensitivity plus specificity (Liu, Pacitti, Valduriez, & Mattoso, 2015). In the final step, only partitions with TSS > 0.7 were selected to create a suitability map by the average of the algorithm combination (Table 1). The entire modeling process was described and performed in ModelR (Sánchez et al., 2018).

**Table 1:** Statistics for each partition of the algorithms used in creating the model

Partition	Algorithm	TSS	Omission	Prevalence	Sensitivity
**1**	**Maxent**	**0.832**	**0.641**	**0.066**	**0.649**
**2**	**Maxent**	**0.757**	**0.384**	**0.032**	**0.39**
**3**	**Maxent**	**0.778**	**0.41**	**0.02**	**0.412**
**1**	**RF**	**0.728**	**0.002**	**0.015**	**0.002**
**2**	**RF**	**0.769**	**0.009**	**0.018**	**0.01**
**3**	**RF**	**0.925**	**0.081**	**0.014**	**0.083**
1	SVM	0.573	−0.01	0.015	−0.01
2	SVM	0.488	0	0.017	0
**3**	**SVM**	**0.964**	**0.013**	**0.014**	**0.014**
1	BioClim	0.373	0	0	0.182
**2**	BioClim	0.61	0	0	0.2
**3**	**BioClim**	**0.758**	**0**	**0**	**0.182**

Note

Partitions used in the final environmental suitability map are in bold.

### 2.3 Environmental suitability and initial establishment

Sowing experiments were used to evaluate initial establishment success and, consequently, to validate the model. Seeds were acquired from ripe fruits collected from at least 10 different adults at each study site (ESP and TPSP) in January 2013, just before the establishment of the experiment. Experimental stations (*n* = 72) were distributed in areas with high (A: 0.89 and B: 0.82) and low (C: 0.22 and D: 0.10) environmental suitability values as determined by the model, with 18 experimental stations for each area. At each station, 20 seeds were placed in the soil spaced 5 cm apart and covered by a cage to exclude vertebrate predators. Monthly visits were made for six consecutive months to assess seed fate as intact, preyed upon by invertebrates or germinated.

The performance of initial establishment in areas of the four levels of environmental suitability was evaluated using a one‐way ANOVA with germination and predation rates as dependent variables. Other one‐way ANOVA was made using microscale variables as dependent variables in order to identify variations in soil characteristics and canopy opening between areas. All statistical tests and graphs were done using R software (R core team, 2017).

### 2.4 Field environmental characterization

The microhabitat of each experimental station was characterized in order to complement the results obtained by the model through understanding environmental conditions present at smaller scales. To evaluate microclimatic conditions throughout the six months of the in situ experiment, relative humidity (%) and average, minimum, and maximum temperatures (°C) were measured using a datalogger (HOBOU23‐002; Onset, MA, USA) in each study area. Soil samples were collected near the experimental stations for analysis of the physical and chemical characteristics of the soil. Canopy openness was estimated based on 18 hemispherical photographs taken in each study area using a Nikon D3100 camera with a fisheye lens (Nikon 10.5 mm) positioned 1 m above the ground. The photographs were analyzed using Gap Light Analyzer (Frazer, Canham, & Lertzman, 1999) after the contrast had been adjusted as necessary.

Data for a total of 17 environmental variables (10 for soil, canopy openness, and six for temperature and humidity) from 72 experimental stations were submitted to logarithmic transformation, and then, a principal component analysis (PCA) was performed in the Vegan package of R software (Dixon, 2003).

## 3 RESULTS

**Figure 1:**
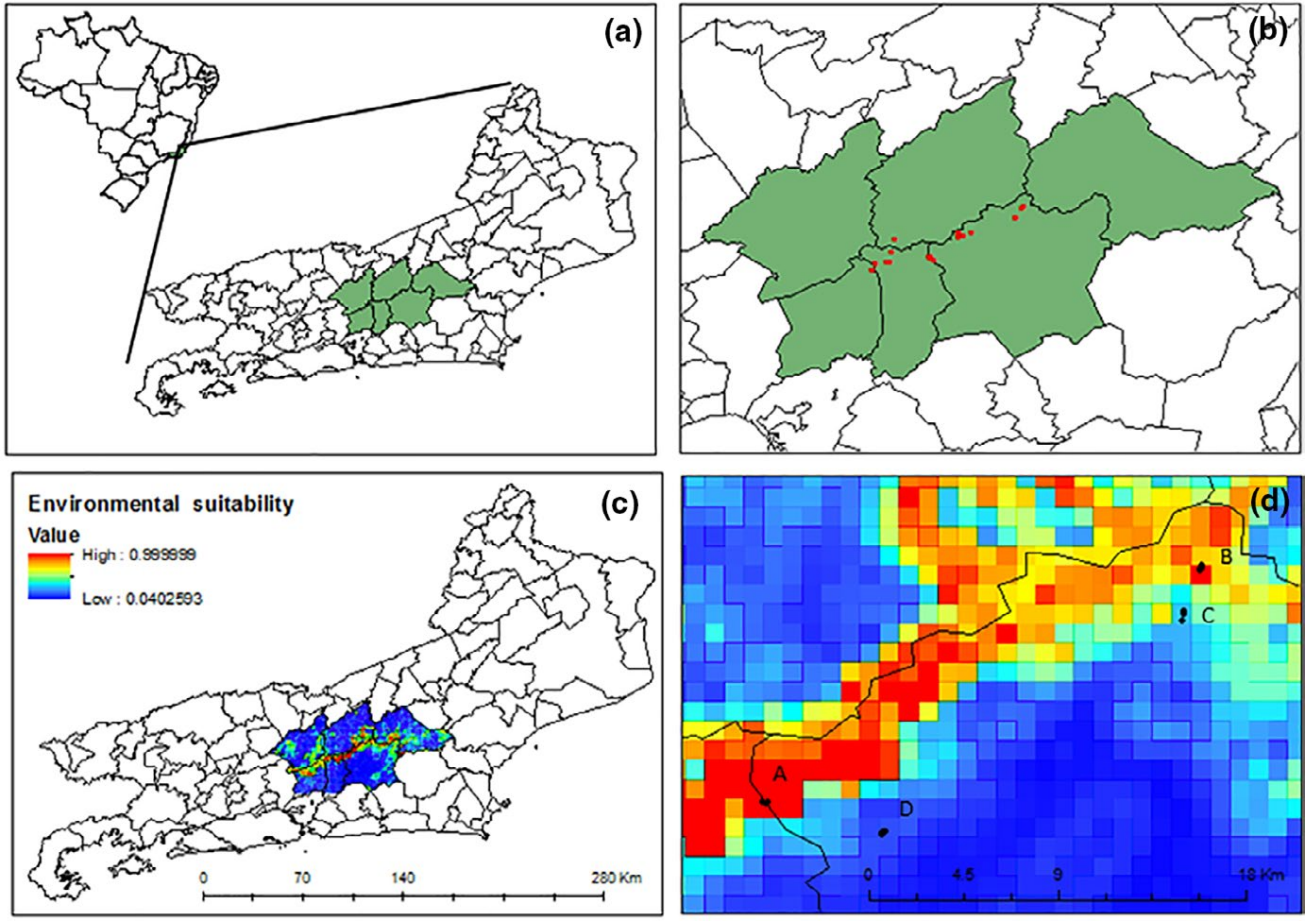
Distribution of *Syagrus weddelliana* (h.Wendl.) Becc. in the State of Rio de Janeiro, Brazil (a). The green area indicates the known area of occurrence for the species, while the red dots indicate records of occurrence (b). Areas of environmental suitability for the species in the study area (c), with experimental stations, located in four areas with different environmental suitability values (in parentheses) represented by black dots: A (0.89), B (0.82), C (0.22), and D (0.10) (d)

The resultant ecological niche models performed fairly well, with some variation among the algorithms used (Table. 1). Some of the maxent partitions had weak abilities to predict true presence and a high variability in the ability to predict true absence (Figure 1).

**Figure 2:**
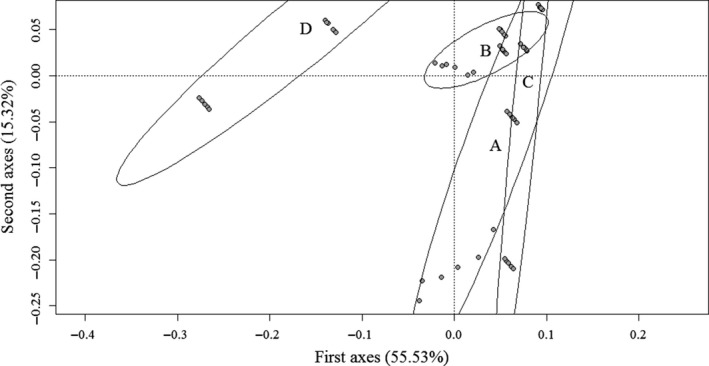
Ordering diagram for the principal components analysis (PCA) of environmental variables measured at experimental stations installed along a gradient of environmental suitability: A (0.89), B (0.82), C (0.22), and D (0.10)

The variables that best explained the areas of higher suitability were those associated with higher relative humidity, sandy soil, and lower canopy openness. The first and second components of the PCA explained 55.53% and 15.32% of the variation among stations, respectively. For the first axis, areas with successful germination were on the right (A, B, and C), while the area that did not show germination was on the left (D) (Figure 2). In general, areas on the right exhibited successful germination and high suitability, with the exception of C, which had low suitability but high germination.

**Figure 3:**
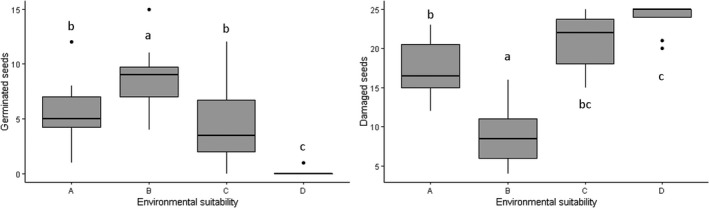
Boxplots with average of germination and predation seeds for the experimental stations installed in areas with different environmental suitability: A (0.89), B (0.82), C (0.22), and D (0.10). Same letters indicate no statistical difference

The number of germinated (*F* = 47.34, *p* < 0.001) and damaged (*F* = 70.43, *p* < 0.001) seeds differed significantly among areas with different environmental suitability (Figure 3). Areas with higher environmental suitability had higher germination and lower predation rates. The area with the lowest suitability (D) had no germination and high rates of predation. The area with the next lowest suitability (C) had some seeds germinated despite high predation. Moreover, in the areas where seeds were attacked the most, fewer seeds germinated (Figure 3). Thus, it seems that abiotic and/or biotic variables directly affect the establishment of *S. weddelliana*.

**Figure 4:**
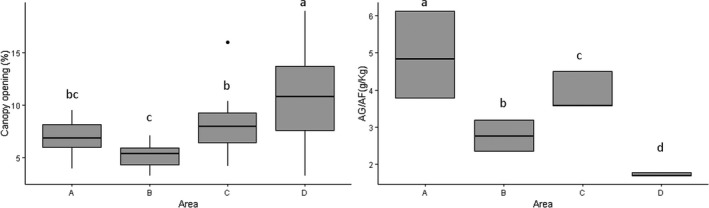
Boxplots with average values of canopy openness and sand granulometry for the experimental stations installed in areas with different environmental suitability values: A (0.89), B (0.82), C (0.22), and D (0.10). Same letters indicate no statistical difference

Despite having low environmental suitability on the macroscale for initial establishment of *S. weddelliana,* the microhabitat of Area C is similar to that of areas with high environmental suitability. The same, however, was not true for microhabitat variables for Area D. This area differed more from the other areas in the percentage of sand (*F* = 106.1, *p* < 0.001) and canopy openness (*F* = 106.1, *p* < 0.001) (Figure 4). These two variables are the ones that better explained the distribution of areas in the first component of the PCA. This demonstrates that, to a certain extent, soil attributes were more important than climate in determining *S weddeliana* distribution. Therefore, soil with thick granulometry and limited canopy openness are important conditions for the initial establishment of *S. weddelliana*.

## 4 DISCUSSION

Our results demonstrated a correspondence among the results of sowing experiments, abiotic and biotic conditions measured in the field, and the ecological niche model. Both variables collected in the field and those obtained in digital databases were complementary and characterized the experimental areas in the same way. In local microscale, soil attributes were more important than climate for explaining areas with and without seed germination. This indicates that the macro‐ and microecological information was complementary and together led to a better understanding of the distribution of *S. weddelliana*. Field validation of ENMs is complicated because different factors and traits of plants can determine their frequency on regional versus local scales (Eiserhardt et al., 2011). While models were constructed using regional temperature and precipitation data, field trials monitored the local climate, which may be quite different (Sheppard, Burns, & Stanley, 2014). Nonetheless, studies have managed to capture the efficiency of ENM even in cases of differences between macro‐ and microhabitat. Field performance has been shown to be highly correlated with ENM; for example, previous studies show good performance in the establishment of exotic species in areas of high environmental suitability outside their natural distribution (Sheppard et al., 2014). The results of the present study show, however, that ENM using occurrence records of adult individuals does not always reflect success in the initial establishment of a species.

In general, areas of higher suitability had successful seed germination while areas with low suitability did not, with the exception of Area C, which, despite having low suitability, had successful seed germination. Initially, germination success in one of the absence areas can be seen as a type I error, that is, an underestimate of the areas of species presence by the model. However, even though this area possesses a microhabitat with adequate conditions (very similar to Areas A and B) for initial establishment, high predation rates can be a limiting factor for the occurrence of *S. weddelliana*. Different factors can act differently during life stages and factors that govern initial establishment vary from those that maintain the persistence of adult individuals in their habitat (Donohue et al., 2010; Spindelböck et al., 2013). Since initial establishment is a critical phase of plant life (Donohue et al., 2010; Spindelböck et al., 2013), germination success in areas with the presence of adult individuals of the same species can be seen as a form of validation of the model. Therefore, it is important to highlight the mortality between seedling to adult stages (Souza, Portela, & De Mattos, 2018) were not evaluated here. So, there is a synergistic interaction between micro‐ and macroscale conditions that influence both initial establishment and older stages.

The influence of microhabitat conditions on the distribution of *S. weddelliana* was expected because the species has a narrow germination niche with respect to water and light availability (Braz, Portela, Cosme, Marques, & Mattos, 2014). Species with restricted distributions are very dependent on the specific conditions of their microhabitat (Peterson, 2003) and less tolerant of environmental change (Cochrane et al., 2015). This is especially important given the current scenario of climate change, because the extent of habitat loss, such as the Atlantic Forest, and the associated extinction risks to endemic species with restricted ranges are poorly understood (Dirnbock, Essl, & Rabitsch, 2011).

In addition to having inadequate micro‐ and macroscale conditions, limitation by predation in Area D was also of great importance. The Enemy Release Hypothesis predicts that a species will be successful in a new habitat when its former enemies (e.g., predators) are absent (Keane & Crawley, 2002). High predation rates were observed in the present study indicating the role of a biotic filter in limiting the establishment of this species in these areas. To confirm this biotic limitation, it would be useful to trace the potential distribution of the main predator of *S. weddelliana*, since climate can also act indirectly through the distribution of its seed predator. However, modeling with terrestrial invertebrates is still very rare especially since museum data are often biased or scarce (Stockman, Beamer, & Bond, 2006).

We conclude that ecological niche models can be used in studies of natural regeneration, but consideration must be given to variation in microhabitat and interactions with other species, especially in absence areas. Biotic factors are important constraints on the distribution of tropical species and should be considered in ENM, especially in small scale studies, to make models more realistic. The ENMs can help even microscale studies, for example, identifying areas of species suitability for the definition of experimental sites. In addition to bringing results complementary to those obtained in microscale. For *S. weddelliana*, which has a restricted distribution, microhabitat conditions, such as canopy openness and edaphic characteristics, appear to be more important for initial establishment than macroclimatic variables. Environmental data collected in the field generated insights about when and how *S. weddelliana* is limited and provided an important contribution to niche model interpretation and validation.

## CONFLICT OF INTEREST

None declared.

## AUTHORS CONTRIBUTIONS

All authors conceived and designed the study; G.A.O. collected the field data; G.A.O. conducted the analyses and wrote the paper with significant contributions from all other authors.

## Data Availability

The data were deposited on Dryad repository and can be accessed at: https://doi.org/10.5061/dryad.m644b82
